# Identification of Hyper-Methylated Tumor Suppressor Genes-Based Diagnostic Panel for Esophageal Squamous Cell Carcinoma (ESCC) in a Chinese Han Population

**DOI:** 10.3389/fgene.2018.00356

**Published:** 2018-09-05

**Authors:** Chenji Wang, Weilin Pu, Dunmei Zhao, Yinghui Zhou, Ting Lu, Sidi Chen, Zhenglei He, Xulong Feng, Ying Wang, Caihua Li, Shilin Li, Li Jin, Shicheng Guo, Jiucun Wang, Minghua Wang

**Affiliations:** ^1^Department of Biochemistry and Molecular Biology, Medical College, Soochow University, Suzhou, China; ^2^State Key Laboratory of Genetic Engineering, Collaborative Innovation Center for Genetics and Development, School of Life Sciences, Fudan University, Shanghai, China; ^3^Human Phenome Institute, Fudan University, Shanghai, China; ^4^Ministry of Education Key Laboratory of Contemporary Anthropology, Department of Anthropology and Human Genetics, School of Life Sciences, Fudan University, Shanghai, China; ^5^Genesky Biotechnologies Inc., Shanghai, China; ^6^Center for Precision Medicine Research, Marshfield Clinic Research Institute, Marshfield, WI, United States

**Keywords:** esophageal squamous cell carcinoma (ESCC), DNA methylation, biomarker, diagnosis, targeted bisulfite sequencing (TGS)

## Abstract

DNA methylation-based biomarkers were suggested to be promising for early cancer diagnosis. However, DNA methylation-based biomarkers for esophageal squamous cell carcinoma (ESCC), especially in Chinese Han populations have not been identified and evaluated quantitatively. Candidate tumor suppressor genes (*N* = 65) were selected through literature searching and four public high-throughput DNA methylation microarray datasets including 136 samples totally were collected for initial confirmation. Targeted bisulfite sequencing was applied in an independent cohort of 94 pairs of ESCC and normal tissues from a Chinese Han population for eventual validation. We applied nine different classification algorithms for the prediction to evaluate to the prediction performance. *ADHFE1, EOMES, SALL1* and *TFPI2* were identified and validated in the ESCC samples from a Chinese Han population. All four candidate regions were validated to be significantly hyper-methylated in ESCC samples through Wilcoxon rank-sum test (*ADHFE1*, *P* = 1.7 × 10^-3^; *EOMES*, *P* = 2.9 × 10^-9^; *SALL1*, *P* = 3.9 × 10^-7^; *TFPI2*, *p* = 3.4 × 10^-6^). Logistic regression based prediction model shown a moderately ESCC classification performance (Sensitivity = 66%, Specificity = 87%, AUC = 0.81). Moreover, advanced classification method had better performances (random forest and naive Bayes). Interestingly, the diagnostic performance could be improved in non-alcohol use subgroup (AUC = 0.84). In conclusion, our data demonstrate the methylation panel of *ADHFE1*, *EOMES*, *SALL1* and *TFPI2* could be an effective methylation-based diagnostic assay for ESCC.

## Background

Esophageal cancer is one of the most aggressive malignant tumors with high prevalence and poor prognosis worldwide (Siegel et al., 2016). Esophageal cancer usually occurs as two subtypes, esophageal squamous cell carcinoma (ESCC) and esophageal adenocarcinoma (EAC), which differed significantly in pathogenesis, pathology, epidemiology and geographical distribution (Enzinger and Mayer, 2003). The regions of the highest occurrence of esophageal cancer stretching from northern China to northwestern Iran, including Japan and India, are localized in the so-called Asian Esophageal Cancer Belt (Kmet and Mahboubi, 1972; Khuroo et al., 1992). The prevalence of ESCC and EAC in these regions are significantly unbalanced with 90% of esophageal cancer patients are ESCCs (Jemal et al., 1972). In addition, the clinical outcomes of ESCC patients depend largely on its diagnosed stage (Enzinger and Mayer, 2003). The majority of ESCCs are diagnosed at advanced stages and the overall 5-year survival rate is relatively poor, while the 5-year survival rate for early stage diagnosed ESCC patients is significantly higher (Besharat et al., 2008). Therefore, it is imperative to identify biomarkers for early diagnosis of ESCC patients.

DNA methylation, which usually occurs in CpG dinucleotides, functioning as an epigenetic modification in mammalian genome and is involved in regulating gene and microRNA expression and alternative splicing. Global hypo-methylation as well as the hyper-methylation of CpG islands in the tumor suppressor genes have been widely identified in the process of tumorigenesis (Baylin et al., 2001). DNA methylation was the first epigenetic alteration to be identified in cancer and multiple lines of studies have found that DNA methylation alterations could serve as biomarkers for cancer diagnosis including ESCC. For example, dozens of genes have been reported to be hyper-methylated in ESCC, including *APC, MGMT, CDH1, RASSF1* (Kawakami et al., 2000; Kuroki et al., 2003; Takeno et al., 2004; Chen et al., 2012). In addition, due to the heterogeneity of ESCC, a single biomarker could only achieve relatively limited prediction ability, which calling for the comprehensive combinations of these candidate biomarkers.

In the present study, we first collected 65 candidate tumor suppressor genes and evaluated their methylation status in ESCC and adjacent control tissues from The Cancer Genome Atlas (TCGA) and Gene Expression Omnibus (GEO) datasets. After a stringent biomarker selection procedure, four of the candidate hyper-methylated genes (*ADHFE1, EOMES, SALL1, TFPI2*) were validated with high-throughput datasets from public databases. Moreover, the methylation profiles of these four genes were further validated with targeted bisulfite sequencing method in 94 pairs of ESCC tumor and adjacent control tissues from a Chinese Han population, yielding a robust performance for ESCC diagnosis.

## Materials and Methods

### Biomarker Selection Based on Publications and Public Datasets

Firstly, Candidate tumor suppressor genes were collected through the keyword matching (“tumor suppressor gene”) with custom script among 91,225 abstract downloaded from PubMed database and manually re-checked (listed in **Supplementary Table 1**). In order to test the methylation status of these 65 candidate genes in ESCC patients, we searched high-throughput microarray datasets in TCGA and GEO database to collect the DNA methylation profiles of the ESCC samples. After stringent quality control, we found that TCGA project has quantified the methylation profiles of 84 ESCC and 3 normal tissues, as well as 78 EAC and 13 normal tissues. Due to the similarities which were shown through PCA analysis between adjacent control tissues from ESCC and EAC, the 13 normal tissues of EAC were included in our combined dataset as controls equally (**Supplementary Figure 1**). In addition, three datasets in GEO database named GSE52826, GSE74693 and GSE79366 were also retrieved, including 26 ESCC and 10 normal tissues. Eventually, 110 ESCC and 26 normal tissues were included from TCGA/GEO for further study. ComBat was applied for removing the batch effect between the different datasets (Leek et al., 2012). Due to the fact that we want to obtain the diagnostic biomarkers which might be applied for liquid biopsy, we then defined the CpG sites with high methylation percent (>0.25) in the ESCCs and relatively lower methylation percent (<0.25) in the adjacent control tissues as the significant CpG sites. Further, it is widely acknowledged that the methylation status of CpG sites was largely variable in different cell types. As a result, we then filtered out the significant CpG sites with high methylation percentage (>0.25) in either peripheral blood mononuclear cells (PBMC, *N* = 111) or peripheral blood leucocytes (PBL, *N* = 527) of the healthy normal samples from the GEO database. The PBMC dataset came from the GSE53045 dataset, and the PBL dataset was the combination of GSE36054 and GSE42861 dataset (Alisch et al., 2012; Liu et al., 2013; Dogan et al., 2014). Moreover, we selected the candidate genes with at least two eligible significant CpG sites for further validation. In summary, six genes were included (*ADHFE1, EOMES, RUNX1, SALL1, TFPI2, WT1*, **Supplementary Table 2**). After that, we designed the primers for these six genes separately and then applied for multiplex PCR system. Due to the GC percent, PolyT and the number of SNPs in the primers of our targeted regions, we only obtained the multiplex PCR system consisting of the four genes including *ADHFE1*, *EOMES*, *SALL1*, *TFPI2* but could not generate enough high quality reads for *RUNX1* and *WT1*. Therefore, these two genes were then discarded for further analysis. Finally, we validated the methylation of these four candidate genes with 94 pairs of Chinese ESCC and control samples (**Table 1**).

**Table 1:** The methylation status of the 6 CpG sites in the TCGA dataset and the validation dataset.

	CpGsite	Gene	Position(hg19)	Relation to CpG_Island	McaM^a^	McoM^a^	*P*-value^b^	Sens^c^	Spec^c^	AUC^c^
TCGA	cg20295442	*ADHFE1*	chr8:67344665	Island	0.26	0.15	0.18	0.42	0.85	0.61
	cg20912169	*ADHFE1*	chr8:67344720	Island	0.26	0.14	0.22	0.46	0.85	0.60
	cg22383888	*EOMES*	chr3:27764816	N_shore	0.53	0.22	**3.10 × 10^-7^**	0.77	0.92	0.87
	cg04550052	*SALL1*	chr16:51184355	Island	0.46	0.22	**7.10 × 10^-5^**	0.79	0.85	0.78
	cg04698114	*SALL1*	chr16:51184379	Island	0.47	0.22	**1.90 × 10^-4^**	0.77	0.85	0.77
	cg12973591	*TFPI2*	chr7:93519473	Island	0.33	0.15	0.06	0.63	0.88	0.65
										
Validation	cg20295442	*ADHFE1*	chr8:67344665	Island	0.18	0.09	**5.10 × 10^-3^**	0.28	0.95	0.63
	cg20912169	*ADHFE1*	chr8:67344720	Island	0.17	0.07	**2.10 × 10^-3^**	0.30	0.94	0.64
	cg22383888	*EOMES*	chr3:27764816	N_shore	0.31	0.11	**3.30 × 10^-9^**	0.55	0.94	0.77
	cg04550052	*SALL1*	chr16:51184355	Island	0.29	0.13	**2.50 × 10^-4^**	0.44	0.91	0.67
	cg04698114	*SALL1*	chr16:51184379	Island	0.34	0.16	**1.10 × 10^-6^**	0.47	0.96	0.72
	cg12973591	*TFPI2*	chr7:93519473	Island	0.25	0.08	**3.30 × 10^-5^**	0.49	0.89	0.69


*^a^McaM represents the mean methylation percentage of the cases, and the McoM represents the mean methylation percentage of the controls. ^b^*P*-value is calculated through the Wilcoxon rank-sum test followed by FDR (false discovery rate) adjustment for multiple correction. ^c^Sens, sensitivity; while Spec, specificity; AUC, area under curve. The sensitivity, specificity as well as the AUC were both with a logistic regression prediction model without adjustment for gender, age and smoking status and alcohol status.*

### Patients and Samples

Esophageal squamous cell carcinoma samples and their paired adjacent control tissues were obtained for validation study from the First Affiliated Hospital of Soochow University and Fourth Military Medical University between the years of 2011 and 2015. All procedures performed in this study were in accordance with the ethical standards of the institutional research committee and with the 1964 Helsinki declaration and its later amendments. The studies were approved by the institutional review boards of Soochow University at Jiangsu Province and Fudan University, Shanghai, China. Written informed consent was obtained from each study subject. In addition, all of the subjects were re-examined and confirmed by professional pathologists for histopathological diagnosis. All tissues were immediately frozen at -80^°^C after surgical resection. Face-to-face interviews were conducted by professional investigators with a comprehensive questionnaire, including clinical information on tobacco smoking, alcohol consumption and family history.

### DNA Extraction, Bisulfite Conversion and Targeted Bisulfite Sequencing

Genomic DNA from ESCC tumor tissue and adjacent control tissue samples were extracted by AllPrep DNA/RNA Mini Kit (Qiagen, Duesseldorf, Germany) according to the manufacturer’s protocols. For methylation analysis, 500 ng genomic DNA was subjected to bisulfite conversion using the EpiTect Fast DNA Bisulfite Kit (Qiagen, Duesseldorf, Germany). A multiplex PCR was performed first with optimized primer sets combination (**Supplementary Table 3**). PCR amplicons were diluted and amplified using indexed primers and the products (170 – 270 bp) were separated by agarose electrophoresis and purified by QIAquick Gel Extraction kit (Qiagen, Duesseldorf, Germany). Libraries from different samples were quantified and pooled together equally, sequenced with the Illumina Hiseq 2000 platform according to the manufacturer’s protocols. BSseeker2 software was utilized for reads mapping and methylation calling (Guo et al., 2013). Samples and CpG sites with high missing rates (>30%) were removed. In order to make sure the reliability of the technique and analysis pipeline, we take LINE-1 as the technical control, whose methylation rate was decreased in cancer tissues compared with normal tissues. Therefore, LINE-1 methylation status was applied to check the credibility of the experiments. Meanwhile, the conversion ratio of C to T in non-CpG sites were applied to evaluate the bisulfite conversion efficiency.

### The 5-aza-2′-deoxycytidine Treatment and Quantitative-PCR

CaEs-17 cells lines were split to low density (25% confluence) per well into 6-well cell culture plates and incubated at 37°C in a humidified incubator with 5% CO2, following culturing overnight. Cells were treated with 5-aza-2′-deoxycytidine (DAC, Sigma, St. Louis, MO, United States) at a concentration of 20 μM in the growth medium, which was exchanged every 24 h for a total of 96 h treatment. After treatment, total RNA was extracted using TRIzol reagent (ThermoFisher, Rockford, IL, United States) from cultured cells. Reverse transcription was performed using 1.5 μg total RNA with an All-in-One cDNA Synthesis SuperMix (Bimake, Houston, TX, United States) according to the manufacturer’s protocol. Meanwhile, qPCR was used to detect the expression of SALL1, EOMES, TFPI2, ADHFE1 mRNA in a reaction volume of 10 μl, including 5 μl SYBR Green (Bimake, Houston, TX, United States), 1 μl cDNA, 0.5 μl of each primer and 3 μl water. The mixture was incubated by the following program: 95°C for 5 min, 40 cycles of 95°C for 15 s, 60°C for 1 min. The primers used for reverse transcription was listed in **Supplementary Table 4**.

### Statistical Analysis and Machine Learning

In the first and second stage, we tested the differential methylation of the CpG sites between cancer and normal tissues using Wilcoxon rank-sum test. False discovery rate (FDR) correction was conducted for multiple test correction. In order to discriminate the ESCC tumor and normal tissues, we utilized several machine learning methods, including logistic regression (Package stats), support vector machine (SVM, Package e1071), random forest (Package randomForest), naïve Bayes (Package e1071), neural network (Package nnet), linear discriminant analysis (LDA, Package mda), mixture discriminant analysis (MDA, Package mda), as well as the flexible discriminant analysis (FDA, Package mda) followed with five-fold cross-validation. All statistical analyses were conducted using R 3.2.1 (Dessau and Pipper, 2008).

## Results

### Public Datasets Collection and CpG Sites Validation

**FIGURE 1:**
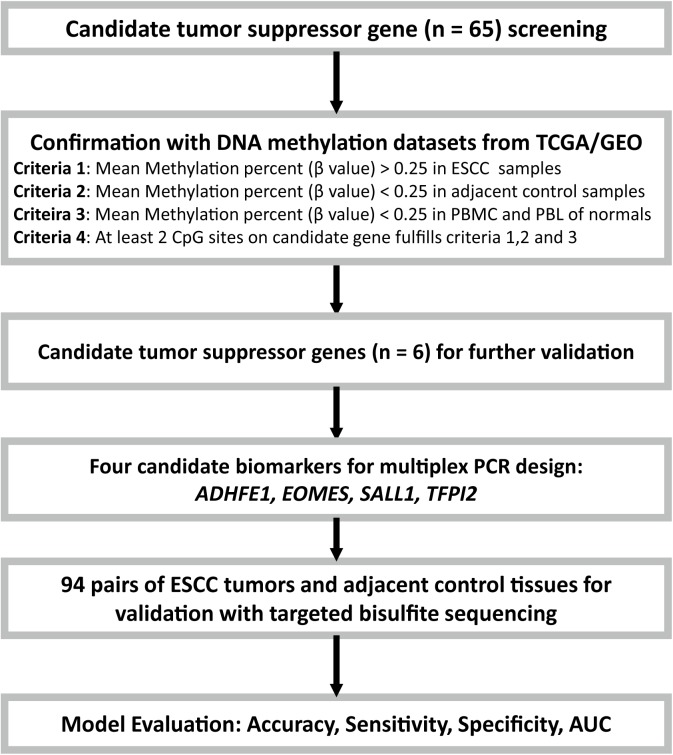
Flow diagram of the study design. Candidate tumor suppressor genes were selected based on literature screening, and their methylation status in ESCC and adjacent control tissues were tested with the ESCC methylation data from the TCGA/GEO datasets. Moreover, the PBMC and PBL methylation datasets from healthy controls from GEO database were also included for further confirmation. Finally, due to the limitations of the multiplex PCR design, four of the six candidate tumor suppressor genes were then selected and validated with targeted bisulfite sequencing in an independent Chinese Han ESCC patients.

In order to quantify the methylation status of these four candidate genes, public DNA methylation microarray datasets of ESCC were carefully searched. The detailed biomarker identification procedure was shown in **Figure 1**. In total, 110 ESCC tumor tissues and 26 adjacent control tissues were enrolled (Li et al., 2014; Hao et al., 2016; Kishino et al., 2016). Based on the CpG sites selection criteria which was described in Patients and Methods, six significant CpG sites (cg20295442, cg20912169, cg22383888, cg04550052, cg04698114, cg12973591) located at the four candidate genes were selected for validation (**Table 1**). Integratively, though some of the six CpG sites did not reach the statistical significance threshold due to the limited sample size, we still believed that all of these 6 CpG sites may be of potential as the non-invasive potential biomarkers for ESCC and thus were included for validation. To test the prediction ability based on these six CpG sites, we built a prediction model based on the logistic regression using the methylation status of these 6 CpG sites without adjustment for age, gender and other covariates, which provided a fair good performance to discriminate between ESCC and normal tissues (Sensitivity = 79%, Specificity = 92%, AUC = 0.87). To further evaluate and validate the diagnostic ability of these six CpG sites, we then conducted the validation study in 94 paired ESCC and adjacent control tissue samples obtained from the patients from the Chinese Han population.

### Methylation Status Validation With Targeted Bisulfite Sequencing

**FIGURE 2:**
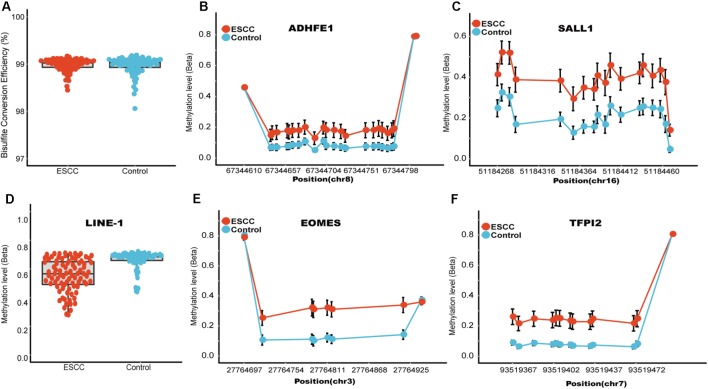
Quality control and the methylation status of these four candidate genomic regions. **(A)** Represent the bisulfite conversion rate calculated by using the number of transformed C to T divided by the number of C of non-CpGs in each sample. **(B)** Represent the methylation status of the technical control LINE-1, which has been shown to be hypo-methylated in several different kinds of tumors. **(C–F)** Represents the CpG sites in regions covering *ADHFE1, EOMES, SALL1, TFPI2*, respectively. The *x* axis represents actual position of each CpG sites in the hg19 reference genome. The *y* axis represents the mean methylation percentage in the ESCC tumor tissues as well as the normal tissues for each of the CpG sites.

**FIGURE 3:**
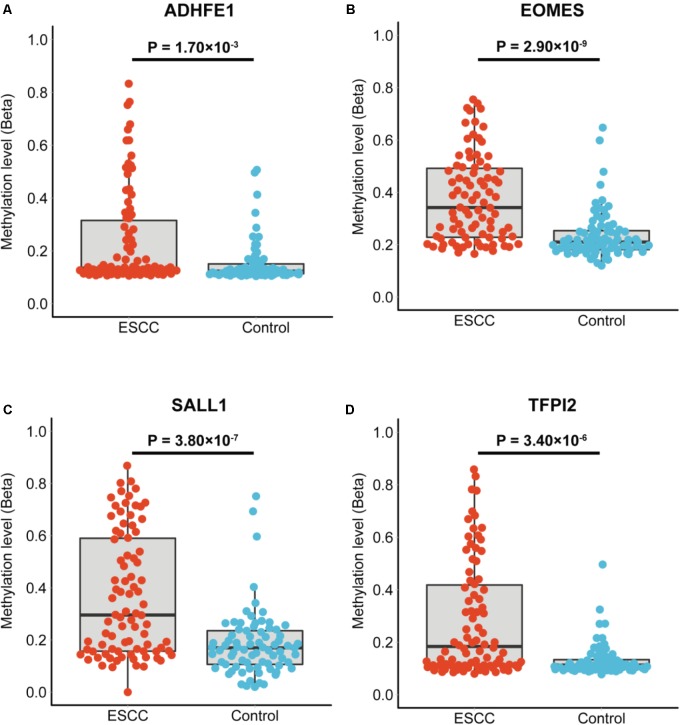
The mean methylation status of each genomic region in tumor and normal tissues**. (A–D)** Represent the mean methylation status of the genomic regions covering *ADHFE1, EOMES, SALL1, TFPI2*, respectively. Each point represents mean methylation percentage in a genomic region of a sample. The boxplot showed the overall methylation percentage of different groups in each genomic region. *P*-value is calculated through the Wilcoxon rank-sum test and the Benjamini-Hochberg procedure was applied for multiple test correction.

The characteristics of the ESCC patients are shown in **Supplementary Table 5** In order to give a robust characterization of the methylation status of these 6 CpG sites as well as the four genes, we applied the targeted bisulfite sequencing method, which was based on the next generation sequencing (NGS) platforms. Because the NGS platforms could generate millions of reads with length > 200 bp, we then designed to test four genomic regions for the four candidate tumor suppressor genes for validation (**Table 2**). In the quality control process, we found that the bisulfite conversion rate (C to T ratio in non-CpG loci) of our samples were higher than 98%, and no significant difference was found between the tumor and adjacent control tissues (**Figure 2A**). Besides, we used the LINE-1 methylation status as technical control and showed that our study was robust and reliable (**Figure 2B**). In addition, the samples and the CpG sites with high missing rates were also filtered out as described in Patients and Methods. After quality control, 163 samples remained for further study. PCA analysis revealed that a significant distinction between ESCC samples and control samples (**Supplementary Figure 2**). Differential methylation analyses were conducted for the four genomic regions, suggesting a major difference between the ESCC and adjacent control tissues (**Figures 2C–F**). A logistic regression model was then applied, and showed significant hyper-methylation status of the six selected CpG sites in the ESCC tissues (**Table 1**, cg20295442, *p* = 5.10 × 10^-3^; cg20912169, *p* = 2.10 × 10^-3^; cg22383888, *p* = 3.30 × 10^-9^; cg04550052, *p* = 2.50 × 10^-4^; cg04698114, *p* = 1.10 × 10^-6^; cg12973591, *p* = 3.30 × 10^-5^). To better characterize the methylation status of the four genomic regions as well as the four candidate genes, we averaged the methylation status of all the CpG sites in each genomic region and conducted the DMR analysis with the same approach. We found all these 4 genes are significantly differentially methylated between ESCC and normal samples (**Figure 3**). Based on the mean methylation status of the four genomic regions, the prediction ability of each region separately was evaluated through logistic regression without adjustment for age, gender and other covariates. The sensitivity of each region ranges from 29 to 69%, while the specificity ranges from 77 to 94%, and the AUC ranges from 0.64 to 0.78 (**Table 2**). Of these four candidates, *EOMES* showed the highest sensitivity (0.69) and AUC (0.78), while the *ADHFE1* showed the best specificity (0.94). Moreover, in the logistic model taking all of the four regions as predictors, we obtained the sensitivity of 66% and specificity of 87%, as well as the AUC of 0.81 (**Supplementary Figure 3**).

**Table 2:** The mean methylation status of the 4 genomic regions in the validation datasets.

Genomic Region^a^	No. CpG sites^b^	CpGsite Included	Gene	McaM^c^	McoM^c^	*P*-value^d^	log_10_(OR)^e^	95% CI^e^	Sens^f^	Spec^f^	AUC^f^
chr8:67344610-67344805	24	cg20295442, cg20912169	*ADHFE1*	0.24	0.15	**1.70 × 10^-3^**	2.20	1.00–3.72	0.29	0.94	0.64
chr3:27764697-27764940	8	cg22383888	*EOMES*	0.38	0.24	**2.90 × 10^-9^**	3.88	2.51–5.51	0.69	0.77	0.78
chr16:51184268-51184468	18	cg04550052, cg04698114	*SALL1*	0.37	0.19	**3.90 × 10^-7^**	2.41	1.51–3.51	0.53	0.90	0.74
chr7:93519367-93519503	13	cg12973591	*TFPI2*	0.28	0.13	**3.40 × 10^-6^**	3.82	2.26–5.89	0.50	0.91	0.71


*^*a*^Genomic region represents the genomic coverage of the reads with targeted bisulfite sequencing, and the genomic coordinates shown here is based on the hg19 version of the genome. ^*b*^No. CpG sites represents the number of the CpG sites in each region. ^*c*^McaM represents the mean methylation percentage of the cases in each region, which consisting of several CpG sites, while the McoM represents the mean methylation percentage of the controls in each region. ^*d*^P value is calculated through the Wilcoxon rank-sum test following with FDR (false discovery rate) adjustment for multiple correction. ^*e*^OR and 95% CI were conducted through logistic regression. ^*f*^Sens, sensitivity; while Spec, specificity; AUC, area under curve. The sensitivity, specificity as well as the AUC were both with a logistic regression prediction model without adjustment for gender, age and smoking status and alcohol status.*

### The Prediction Performance of the Diagnosis Panel in Different Classification Models

Several machine learning methods, including logistic regression model, random forest, support vector machine (SVM), neural network (NN), Naïve Bayes (NB), linear discriminant analysis (LDA), mixture discriminant analysis (MDA), flexible discriminant analysis (FDA), and gradient boosting machine (GBM) following with fivefold cross validation were utilized for ESCC classification based on the targeted bisulfite sequencing regions (**Table 3**). It turned out that the GBM model achieved the highest classification accuracy among all machine learning methods in train stage, whose sensitivity, specificity and accuracy were 82.6, 85.6, and 84.0%. The Naive Bayes model achieved the best specificity (91.6%) in the train stage. In the test stage, the random forest and Naive Bayes performed with the best sensitivity (72.8%) and specificity (91.0%), respectively. In addition, the linear discriminant analysis and flexible discriminant analysis model both achieved the best accuracy (73.5%).

**Table 3:** Diagnosis accuracy, sensitivity and specificity of different classification models with fivefold cross-validation.

Methods	Train			Test		
		
	Sensitivity	Specificity	Accuracy	Sensitivity	Specificity	Accuracy
Logistic Regression	0.683	0.873	0.773	0.645	0.830	0.732
Random Forest	0.726	0.739	0.732	**0.728**	0.741	0.734
Supporting Vector Machine	0.635	0.907	0.764	0.599	0.881	0.731
Naive Bayes	0.539	**0.916**	0.718	0.532	**0.910**	0.709
Neural Network	0.701	0.841	0.768	0.667	0.794	0.726
Linear Discriminant Analysis	0.617	0.906	0.754	0.594	0.894	**0.735**
Mixture Discriminant Analysis	0.618	0.868	0.736	0.564	0.843	0.695
Flexible Discriminant Analysis	0.616	0.907	0.754	0.594	0.894	**0.735**
Gradient Boosting Machine	**0.826**	0.856	**0.840**	0.699	0.728	0.713


*The mean methylation percentage of each genomic region was considered as the independent variable for constructing the models, which means that all of the models were based on these five independent variables without adjustment for gender, age, smoking status and alcohol status. Sensitivity, specificity and classification accuracy were the mean value in fivefold cross-validations with 1,000 replications.*

### The Diagnostic Ability in the ESCC Subgroups

Previous studies have found several risk factors for the incidence of ESCC, including age, gender, smoking status, and alcohol status (Wang et al., 2007; Pandeya et al., 2009; Toh et al., 2010). In order to explore the effects of these risk factors on the ESCC diagnosis, we conducted the subgroup analyses. Similarly, the mean methylation percentage of each genomic region was utilized. To explore the diagnostic ability in the young/old samples, we first divided the samples according to the median age of our patients. No significant difference between the sensitivity, specificity and the AUC between the two subgroups (**Supplementary Table 6**). The AUCs in the two subgroups was 0.82 and 0.80 for the young and old subgroups, respectively (**Supplementary Figures 4A,B**). When it comes to the gender, the difference was still quite limited (AUC: 0.79 vs. 0.82 for male and female subgroups, **Supplementary Table 7**). Similarly, no significant difference of the diagnostic performances was found between smoker/non-smoker subgroup analysis (**Supplementary Table 8**). However, when concentrating on the effect of alcohol use, we found that the non-alcohol use subgroup showed obviously higher AUC than that of the alcohol use subgroup (0.84 vs. 0.77 respectively, **Supplementary Table 9**). The significant difference in the diagnostic performance between the alcohol use and non-alcohol use subgroup indicates that alcohol use may contribute to the epigenetic changes in ESCC as well as to the pathogenesis of ESCC (**Supplementary Figures 4C–H**).

### The Association Between Gene Expression and Methylation of the Candidate Genes

**FIGURE 4:**
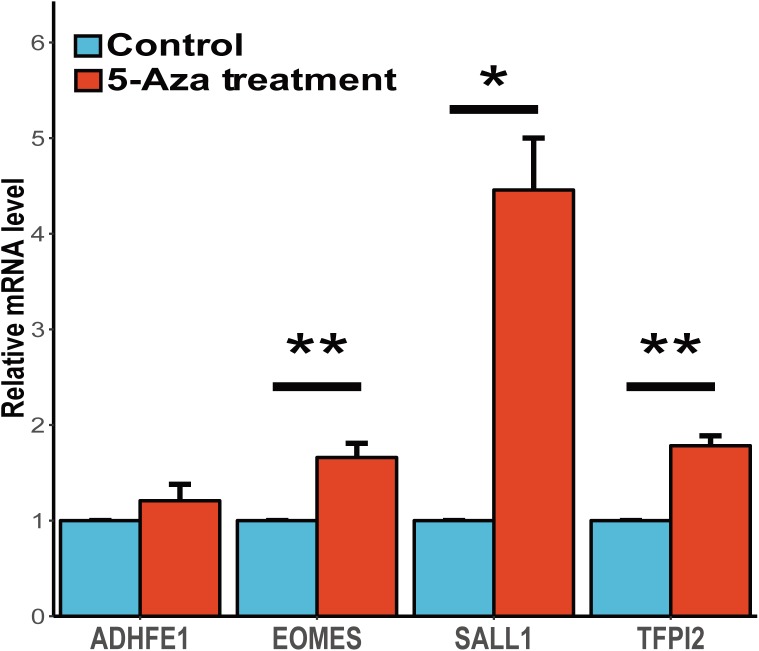
Gene expression change of candidate genes after the treatment of 5-aza-2**′**-deoxycytidine. The expression profiles of these four genes before and after 5-Aza treatment in CaES-17 cell line was shown. The RNA quantification was conducted at three replicates for each gene and the GAPDH mRNA levels were used as an internal standard. The 2^-ΔΔCq^ method was used to analyze the relative changes in these four genes. The Student’s *t*-test was carried out to test the differential expression after the 5-Aza treatment. ^∗^Indicates *P* < 0.05, ^∗∗^indicates *P* < 0.01 while ^∗∗^indicates *P* < 0.001.

It is widely accepted that the gene methylation could regulate the gene expression level and further affect the physiological activities. To assess the associations between gene expression and methylation of these four candidates, we conducted the study to demethylase the human esophageal squamous carcinoma cell line (CaES-17) with 5-aza-2′-deoxycytidine and quantified the gene expression of these candidate genes. We found three of these four genes (*EOMES, SALL1 and TFPI2*) shown a significant up-regulation after 5-aza-2′-deoxycytidine treatment, while *ADHFE1* showed a slight up-regulation yet the statistic test was not quite significant (**Figure 4**). In summary, our results validated the inverse correlations between gene expression and methylation of these four genes, and suggesting that abnormal methylation change of these genes might be involved in ESCC carcinogenesis mediated by gene expression change.

## Discussion

In this study, 4 out of 65 candidate tumor suppressor genes (*ADHFE1, EOMES, SALL1, TFPI2*) were found to be hyper-methylated in ESCC tissues while hypo-methylated in the adjacent control tissues as well as the peripheral blood samples, and were further validated in an independent 94 pairs of ESCC and adjacent control tissues from Chinese Han population.

Of these four candidate genes, alcohol dehydrogenase, iron containing 1 (*ADHFE1*) encodes hydroxyacid-oxoacid transhydrogenase, which is responsible for the oxidation of 4-hydroxybutyrate in mammalian tissues (Kardon et al., 2006). *ADHFE1* promoter hyper-methylation was found in colorectal cancer (CRC) and the alcohol could down-regulate the expression of *ADHFE1* through hyper-methylation and further induce the proliferation of CRC cells (Tae et al., 2013; Moon et al., 2014). Meanwhile, Xi et al. also identified that *ADHFE1* was one of the target genes of differentially expressed miRNAs in esophageal adenocarcinomas (Xi and Zhang, 2017).

*EOMES* belongs to the TBR1 (T-box brain protein 1) sub-family of T-box genes, encoding a transcription factor which is necessary for the embryonic development. It has been reported that *EOMES* promoter methylation could serve as a promising biomarker for the prediction of occurrence, recurrence and prognosis of bladder cancer (Reinert et al., 2011, 2012; Kim et al., 2013). In addition, EOMES has also been confirmed to have potential anti-cancer functions through siRNA experiments, and was regarded as a candidate tumor suppressor gene for human hepatocellular carcinoma (Gao et al., 2014). Spalt like transcription factor 1 (*SALL1*) encodes a zinc finger transcriptional repressor, which has recently been identified as a tumor suppressor gene, whose expression was in positive correlation with *CDH1* and associated with the survival of patients in breast cancer (Wolf et al., 2014). In addition, *SALL1* hyper-methylation has already been confirmed as the diagnostic biomarker for breast cancer and other epithelial cancers, especially for the colorectal cancer (Hill et al., 2010).

Tissue factor pathway inhibitor 2 (*TFPI2*) encodes a member of the Kunitz-type serine proteinase inhibitor family, and was found to be down-regulated in 75% of esophageal carcinomas and in most esophageal carcinoma cell lines (Ran et al., 2009). Moreover, Jia et al. (2012) have found that the *TFPI2* is frequently methylated in esophageal cancer with a progression tendency, and the restoration of *TFPI2* expression could inhibit the invasion, migration, colony formation and proliferation in KYSE70 cell line. Therefore, multiple studies have incorporated *TFPI2* into the DNA methylation-based diagnostic panel for ESCC early diagnosis (Corrie et al., 2009; Tsunoda et al., 2009). Similarly, Chettouh et al. (2017) also showed that the methylation status of *TFPI2* promoter could detect Barrett’s esophagus when applied to Cytosponge samples (Chettouh et al., 2017). Moreover, Liu et al. also revealed that celecoxib, which was reported to induce promoter demethylation and reactivate expression of some metastasis-suppressor genes in lung cancer cells, could demethylate the methylation status of *TFPI2 in vivo* and up-regulate the gene expression as well as inducing the apoptosis of cancer cells (Liu et al., 2016). Therefore, the DNA methylation status of *TFPI2* may also be implicated in ESCC treatment.

The accurate early diagnosis of cancer is a great challenge due to the cancer heterogeneity. In our study, we selected four candidate tumorigenesis genes and applied the targeted bisulfite sequencing method to explore the methylation status of our candidate CpG sites as well as their adjacent genomic regions, thus yielding a robust estimation of the methylation status of the candidate genes. With the fast development of NGS technology, the targeted bisulfite sequencing method is becoming more and more popular for methylation detection because of high accuracy, high-throughput and cost-effective. In the past studies, we found the single DNA methylation biomarker usually cannot provide enough prediction power in cancer diagnosis. According to our results, the panel consisting of these four candidate genes could distinguish the ESCC tumors with higher specificity and sensitivity compared with single biomarker.

In summary, a panel with four genes was identified and achieved a fair good accuracy in classifying ESCC from normal tissues. However, according to diagnosis performance, our prediction model still has more space to be improved when we introduce more biomarkers. Multi-omics datasets, including genomics, epigenomics and proteomics, which could provide biomarkers in different biological layers, could contribute to the accurate non-invasive diagnosis of ESCC in the future. In addition, the diagnostic ability of our panel was only validated in ESCC samples but not in EAC samples due to our limited samples, and further studies based on EAC samples should be conducted.

## Conclusion

Integrated analysis of public literatures and multiples high-throughput DNA methylation microarray datasets were conducted and discovered four tumor suppressor genes (*ADHFE1, EOMES, SALL1, TFPI2*) as the candidate biomarkers for ESCC diagnosis. All four tumor suppressor genes were then successfully validated in an independent cohort including 94 pairs of ESCC and adjacent control tissues. Moreover, the EOMES showed the highest sensitivity (0.69) and AUC (0.78), while the ADHFE1 showed the best specificity (0.94). Methylation profiles of *ADHFE1, EOMES, SALL1, TFPI2* could be an effective methylation-based assay (Sensitivity = 0.66, Specificity = 0.87, AUC = 0.81) for the ESCC diagnosis with high specificity.

## Availability of Data and Materials

The datasets used and analyzed in this study have been submitted to European Genome-phenome Archive with the accession number EGAS00001003158.

## Author Contributions

MW, JW, LJ, YZ, and SG contributed to the conception and design of the study. CW, DZ, ZH, and XF contributed to the sample collection and DNA extraction. YW and CL conducted the targeted bisulfite sequencing experiments for the validation stage. WP, SC, and CW contributed to TCGA and GEO as well as the targeted bisulfite sequencing data analysis. WP, MW, JW, and SG wrote the manuscript. All the authors read and approved the final manuscript.

## Conflict of Interest Statement

YW and CL were employed by Genesky Biotechnologies Inc., Shanghai. The remaining authors declare that the research was conducted in the absence of any commercial or financial relationships that could be construed as a potential conflict of interest.
