# Supplementary material for: Identification of Hyper-Methylated Tumor Suppressor Genes-Based Diagnostic Panel for Esophageal Squamous Cell Carcinoma (ESCC) in a Chinese Han Population
Source: Front Genet. 2018 Sep 5;9:356. doi: 10.3389/fgene.2018.00356 (PMC6133993; doi:10.3389/fgene.2018.00356)
Supplement: Supplementary file 2 [file Data_Sheet_2.DOCX]

**Supplementary Table 1 The methylation status of the CpG sites of the candidate tumor suppressor genes in the TCGA/GEO combined dataset**

Each row represents the methylation profiles of a CpG site located at the candidate tumor suppressor genes. McaM represents the mean methylation level (β value) of this CpG site in the ESCC tumor tissues, and McoM represents the mean methylation level in the adjacent normal tissues. P-value was calculated through Wilcoxon rank-sum test and FDR was calculated through the Benjamini-Hochberg procedure. Sens (sensitivity), Spec(specificity) and AUC (area under curve) were all calculated through the logistic regression. In addition, the mean methylation level of the CpG site in PBL (peripheral blood leucocytes) and in PBMC (peripheral blood mononuclear cells) or control samples were also shown.

**Supplementary Table 2 The methylation status of the candidate significant CpG sites in the combined datasets of TCGA/GEO ESCC samples along with the PBMC and PBL datasets from healthy samples**

| **Gene** | **Chr** | **Pos** | **McaM^a^** | **McoM^b^** | **Pvalue^c^** | **FDR^d^** | **Mean.PBL^e^** | **Mean.PBMC^f^** |
| --- | --- | --- | --- | --- | --- | --- | --- | --- |
| EOMES-cg22383888 | 3 | 27764816 | 0.53 | 0.22 | 7.00E-09 | 3.10E-07 | 0.15 | 0.12 |
| EOMES-cg02694810 | 3 | 27764660 | 0.35 | 0.16 | 2.80E-05 | 2.30E-04 | 0.09 | 0.09 |
| EOMES-cg06014401 | 3 | 27765232 | 0.44 | 0.19 | 2.10E-09 | 1.80E-07 | 0.08 | 0.05 |
| WT1-cg05940984 | 11 | 32448678 | 0.36 | 0.16 | 8.00E-05 | 5.50E-04 | 0.06 | 0.07 |
| WT1-cg25094569 | 11 | 32448769 | 0.34 | 0.21 | 2.30E-03 | 9.40E-03 | 0.13 | 0.12 |
| WT1-cg22511262 | 11 | 32455192 | 0.31 | 0.18 | 6.90E-04 | 3.40E-03 | 0.15 | 0.12 |
| WT1-cg16092786 | 11 | 32455735 | 0.37 | 0.23 | 7.00E-05 | 4.80E-04 | 0.13 | 0.11 |
| RUNX1-cg05000748 | 21 | 36399146 | 0.42 | 0.23 | 8.10E-07 | 1.40E-05 | 0.03 | 0.02 |
| RUNX1-cg04228935 | 21 | 36399258 | 0.46 | 0.19 | 2.70E-09 | 2.00E-07 | 0.05 | 0.02 |
| SALL1-cg04550052 | 16 | 51184355 | 0.46 | 0.22 | 6.70E-06 | 7.10E-05 | 0.14 | 0.14 |
| SALL1-cg04698114 | 16 | 51184379 | 0.47 | 0.22 | 2.30E-05 | 1.90E-04 | 0.06 | 0.06 |
| SALL1-cg08526074 | 16 | 51184562 | 0.27 | 0.12 | 1.70E-02 | 5.00E-02 | 0.04 | 0.04 |
| SALL1-cg02864757 | 16 | 51184583 | 0.26 | 0.16 | 2.20E-03 | 9.20E-03 | 0.12 | 0.10 |
| SALL1-cg09016242 | 16 | 51185110 | 0.36 | 0.21 | 3.00E-02 | 8.00E-02 | 0.14 | 0.12 |
| SALL1-cg00310215 | 16 | 51185346 | 0.29 | 0.19 | 8.10E-02 | 1.70E-01 | 0.13 | 0.15 |
| SALL1-cg06724588 | 16 | 51185459 | 0.27 | 0.18 | 7.70E-02 | 1.60E-01 | 0.11 | 0.14 |
| ADHFE1-cg01588438 | 8 | 67344553 | 0.29 | 0.19 | 6.90E-02 | 1.50E-01 | 0.11 | 0.08 |
| ADHFE1-cg09383816 | 8 | 67344556 | 0.25 | 0.15 | 4.50E-02 | 1.10E-01 | 0.10 | 0.06 |
| ADHFE1-cg20295442 | 8 | 67344665 | 0.26 | 0.15 | 9.00E-02 | 1.80E-01 | 0.06 | 0.05 |
| ADHFE1-cg20912169 | 8 | 67344720 | 0.26 | 0.14 | 1.20E-01 | 2.20E-01 | 0.05 | 0.03 |
| ADHFE1-cg01988129 | 8 | 67344936 | 0.33 | 0.21 | 8.00E-03 | 2.60E-02 | 0.14 | 0.12 |
| ADHFE1-cg25046651 | 8 | 67345006 | 0.29 | 0.18 | 4.30E-03 | 1.50E-02 | 0.14 | 0.11 |
| TFPI2-cg12973591 | 7 | 93519473 | 0.33 | 0.15 | 2.00E-02 | 5.70E-02 | 0.07 | 0.06 |
| TFPI2-cg22799321 | 7 | 93519621 | 0.32 | 0.18 | 3.70E-02 | 9.30E-02 | 0.12 | 0.09 |
| TFPI2-cg20230721 | 7 | 93519855 | 0.30 | 0.14 | 2.00E-01 | 3.40E-01 | 0.05 | 0.04 |
| TFPI2-cg23141855 | 7 | 93519892 | 0.25 | 0.17 | 5.60E-01 | 7.00E-01 | 0.13 | 0.10 |

McaM^a^ represent the mean methylation percentage of the ESCC samples in the TCGA/GEO combined dataset, and the McoM^b^ represent the mean methylation percentage of the control samples in the combined dataset. The Pvalue^c^ is calculated through the Wilcoxon rank-sum test. FDR^d^ represents the p-values after multiple test correction with FDR. Mean.PBL^e^ represent the mean methylation percentage of the PBL of normal samples. Mean.PBMC^f^ represents the methylation data of the PBMC of the healthy normal samples. PBL and PBMC datasets were obtained from the public dataset from the GEO database.

**Supplementary Table 3 The designed primers of the five genomic regions for targeted bisulfite sequencing**

| **Primer Name** | **Sequence** |
| --- | --- |
| ADHFE1_F | GTTTTGAGTTYGATTGGTTTGAGG |
| ADHFE1_R | CTACRCRTTACAATTACCTCAACAAATAC |
| EOMES_F | AGTTGTGTTGGYGTGAGTATGAAG |
| EOMES_R | TTCAAAACACACCTTCCTCTTATCRAAAACA |
| SALL1_F | GTAAGAAGATGGGGATTGGTGT |
| SALL1_R | CTTCCCTAACCCCCCTAAAA |
| TFPI2_F | GAGGTTTGTTTAATATTTGAGAAAATTTAGG |
| TFPI2_R | AACAACRCCAACAATTTCTACACCTAA |
| LINE-1_F | AGTAGGGYGAGGTATTGTTTTATTTG |
| LINE-1_R | CAAACTACTATACTAACAATCAACRAAATTCC |

**Supplementary Table 4 The designed primers of the four genes and GAPDH for quantitative-PCR**

| **Primer Name** | **Sequence** |
| --- | --- |
| ADHFE1_F | TGCCATTTTTGACTATGAACACTT |
| ADHFE1_R | GACAGCCCTCTTCAGATACTTAGC |
| EOMES_F | CTCCCATGGACCTCCCGAACAA |
| EOMES_R | AGACAGCCGCCTYCGCTTACAA |
| SALL1_F | TGTCAAGTTCCCAGAAATGTTCCA |
| SALL1_R | ATGCCGCCGTTCTGAATGA |
| TFPI2_F | GCCAACAGGAAATAACGCGG |
| TFPI2_R | CAAGCCTCCCAGGTGTAGAA |
| GAPDH_F | TCGGAGTCAACGGATTTGGT |
| GAPDH_R | TTCCCGTTCTCAGCCTTGAC |

**Supplementary Table 5 Characteristics of the ESCC patients included in this study**

| **Characteristics** | **Patient Distribution** |
| --- | --- |
|  | **N = 94** |
| **Age** | 64 (IQR = 57 to 70) |
| **Sex** |  |
| Male | 69 |
| Female | 25 |
| **Smoked^a^** |  |
| Yes | 58 |
| No | 36 |
| **Alcohol use^b^** |  |
| Yes | 34 |
| No | 58 |
| n.a^d^ | 2 |
| **T stage^c^** |  |
| T2 | 14 |
| T3 | 72 |
| T4 | 5 |
| n.a | 3 |
| **N stage^c^** |  |
| N0 | 44 |
| N1 | 38 |
| N2 | 7 |
| N3 | 3 |
| n.a | 2 |
| **M stage^c^** |  |
| M0 | 90 |
| M1 | 1 |
| n.a | 3 |

ESCC, esophageal squamous cell carcinoma; ^a^Yes represent the former and current smokers. ^b^Yes represent individuals who presently consume or formerly consumed alcoholic beverages. ^c^TNM Stages were assessed by the seventh edition of the TNM classification criteria. The n.a^d^ represents the number of samples with missing values.

**Supplementary Table 6 The methylation status of the 4 genomic regions in the Young/Old subgroups**

| **Subgroup** | **Gene** | **Genomic** | **No.** | **McaM^d^** | **McoM^d^** | **Pvalue^e^** | **Sens^f^** | **Spec^f^** | **AUC^f^** |
| --- | --- | --- | --- | --- | --- | --- | --- | --- | --- |
|  |  | **Region^b^** | **CpGsites^c^** |  |  |  |  |  |  |
| Young^a^  (N =45) | ADHFE1 | chr8:67344610-67344805 | 24 | 0.24 | 0.15 | 7.51×10^-3^ | 0.41 | 0.94 | 0.68 |
|  | EOMES | chr3:27764697-27764940 | 8 | 0.37 | 0.24 | 2.70×10^-4^ | 0.51 | 0.97 | 0.75 |
|  | SALL1 | chr16:51184268-51184468 | 18 | 0.35 | 0.17 | 1.00×10^-4^ | 0.51 | 0.94 | 0.77 |
|  | TFPI2 | chr7:93519367-93519503 | 13 | 0.24 | 0.13 | 6.79×10^-3^ | 0.54 | 0.94 | 0.69 |
|  | Combined | - | - | - | - | - | **0.74** | **0.80** | **0.82** |
| Old^a^  (N = 49) | ADHFE1 | chr8:67344610-67344805 | 24 | 0.23 | 0.15 | 6.54×10^-2^ | 0.26 | 0.95 | 0.61 |
|  | EOMES | chr3:27764697-27764940 | 8 | 0.38 | 0.23 | 6.00×10^-7^ | 0.81 | 0.70 | 0.80 |
|  | SALL1 | chr16:51184268-51184468 | 18 | 0.39 | 0.20 | 3.02×10^-4^ | 0.49 | 0.93 | 0.72 |
|  | TFPI2 | chr7:93519367-93519503 | 13 | 0.32 | 0.14 | 8.87×10^-5^ | 0.43 | 1.00 | 0.74 |
|  | Combined | - | - | - | - | - | **0.68** | **0.88** | **0.80** |

^a^The median ages of the patients were utilized as the criteria for dividing samples into the young and old groups. ^b^Genomic region represents the genomic coverage of the reads with targeted bisulfite sequencing, and the genomic coordinates shown here were based on the hg19 version of the genome. ^c^No.CpGsites represents the number of the CpGsites in each region. ^d^McaM represents the mean methylation percentage of the cases in each region, which consists of several CpGsites, while McoM represents the mean methylation percentage of the controls in each region. ^e^P value is calculated through the Wilcoxon rank-sum test following with FDR (false discovery rate) adjustment for multiple correction. ^f^Sens is short for sensitivty, while Spec is short for specificity, and the AUC is short for area under curve. The sensitivity, specificity as well as the AUC were both with a logistic regression prediction model without adjustment for gender, age and smoking status and alcohol status. The mean methylation percentage of each in each genomic region was considered as a variable.

**Supplementary Table 7 The methylation status of the 4 genomic regions in the Male/Female subgroups**

| **Subgroup** | **Gene** | **Genomic** | **No.** | **McaM^c^** | **McoM^c^** | **Pvalue^d^** | **Sens^e^** | **Spec^e^** | **AUC^e^** |
| --- | --- | --- | --- | --- | --- | --- | --- | --- | --- |
|  |  | **Region^a^** | **CpGsites^b^** |  |  |  |  |  |  |
| Male  (N =69) | ADHFE1 | chr8:67344610-67344805 | 24 | 0.24 | 0.16 | 4.54×10^-3^ | 0.56 | 0.69 | 0.65 |
|  | EOMES | chr3:27764697-27764940 | 8 | 0.37 | 0.24 | 2.40×10^-6^ | 0.65 | 0.76 | 0.76 |
|  | SALL1 | chr16:51184268-51184468 | 18 | 0.36 | 0.18 | 3.13×10^-5^ | 0.48 | 0.95 | 0.73 |
|  | TFPI2 | chr7:93519367-93519503 | 13 | 0.28 | 0.13 | 5.72×10^-5^ | 0.54 | 0.95 | 0.72 |
|  | Combined | - | - | - | - | - | **0.68** | **0.86** | **0.79** |
| Female  (N = 25) | ADHFE1 | chr8:67344610-67344805 | 24 | 0.24 | 0.13 | 1.88×10^-1^ | 0.30 | 1.00 | 0.62 |
|  | EOMES | chr3:27764697-27764940 | 8 | 0.41 | 0.23 | 1.20×10^-3^ | 0.65 | 0.95 | 0.83 |
|  | SALL1 | chr16:51184268-51184468 | 18 | 0.39 | 0.18 | 7.10×10^-3^ | 0.61 | 0.95 | 0.76 |
|  | TFPI2 | chr7:93519367-93519503 | 13 | 0.28 | 0.13 | 1.43×10^-2^ | 0.78 | 0.58 | 0.73 |
|  | Combined | - | - | - | - | - | **0.70** | **0.95** | **0.82** |

^a^Genomic region represents the genomic coverage of the reads with targeted bisulfite sequencing, and the genomic coordinates shown here were based on the hg19 version of the genome. ^b^No.CpGsites represents the number of the CpGsites in each region. ^c^McaM represents the mean methylation percentage of the cases in each region, which consists of several CpGsites, while McoM represents the mean methylation percentage of the controls in each region. ^d^P value is calculated through the Wilcoxon rank-sum test following with FDR (false discovery rate) adjustment for multiple correction. ^e^Sens is short for sensitivty, while Spec is short for specificity, and the AUC is short for area under curve. The sensitivity, specificity as well as the AUC were both with a logistic regression prediction model without adjustment for gender, age and smoking status and alcohol status. The mean methylation percentage of each in each genomic region was considered as a variable.

**Supplementary Table 8 The methylation status of the 4 genomic regions in the Smokers/Non-smokers subgroups**

| **Subgroup** | **Gene** | **Genomic** | **No.** | **McaM^d^** | **McoM^d^** | **P value^e^** | **Sens^f^** | **Spec^f^** | **AUC^f^** |
| --- | --- | --- | --- | --- | --- | --- | --- | --- | --- |
|  |  | **Region^b^** | **CpGsites^c^** |  |  |  |  |  |  |
| Smokers^a^  (N =58) | ADHFE1 | chr8:67344610-67344805 | 24 | 0.23 | 0.16 | 1.61×10^-2^ | 0.60 | 0.64 | 0.64 |
|  | EOMES | chr3:27764697-27764940 | 8 | 0.38 | 0.23 | 7.80×10^-6^ | 0.68 | 0.79 | 0.78 |
|  | SALL1 | chr16:51184268-51184468 | 18 | 0.37 | 0.18 | 3.44×10^-4^ | 0.51 | 0.96 | 0.72 |
|  | TFPI2 | chr7:93519367-93519503 | 13 | 0.30 | 0.14 | 8.39×10^-4^ | 0.57 | 0.94 | 0.70 |
|  | Combined | - | - | - | - | - | **0.64** | **0.94** | **0.80** |
| Non-smokers^a^  (N = 36) | ADHFE1 | chr8:67344610-67344805 | 24 | 0.26 | 0.13 | 6.71×10^-2^ | 0.30 | 1.00 | 0.63 |
|  | EOMES | chr3:27764697-27764940 | 8 | 0.38 | 0.24 | 3.50×10^-4^ | 0.61 | 0.87 | 0.79 |
|  | SALL1 | chr16:51184268-51184468 | 18 | 0.36 | 0.18 | 7.30×10^-4^ | 0.55 | 0.87 | 0.75 |
|  | TFPI2 | chr7:93519367-93519503 | 13 | 0.25 | 0.13 | 7.30×10^-4^ | 0.85 | 0.57 | 0.75 |
|  | Combined | - | - | - | - | - | **0.70** | **0.80** | **0.81** |

^a^The smokers subgroup included the former smokers as well as current smokers. ^b^Genomic region represents the genomic coverage of the reads with targeted bisulfite sequencing, and the genomic coordinates shown here were based on the hg19 version of the genome. ^c^No.CpGsites represents the number of the CpGsites in each region. ^d^McaM represents the mean methylation percentage of the cases in each region, which consists of several CpGsites, while McoM represents the mean methylation percentage of the controls in each region. ^e^P value is calculated through the Wilcoxon rank-sum test following with FDR (false discovery rate) adjustment for multiple correction. ^f^Sens is short for sensitivty, while Spec is short for specificity, and the AUC is short for area under curve. The sensitivity, specificity as well as the AUC were both with a logistic regression prediction model without adjustment for gender, age and smoking status and alcohol status. The mean methylation percentage of each in each genomic region was considered as a variable.

**Supplementary Table 9 The methylation status of the 4 genomic regions in the Alcohol/ Non-alcohol subgroups**

| **Subgroup** | **Gene** | **Genomic** | **No.** | **McaM^d^** | **McoM^d^** | **P value^e^** | **Sens^f^** | **Spec^f^** | **AUC^f^** |
| --- | --- | --- | --- | --- | --- | --- | --- | --- | --- |
|  |  | **Region^b^** | **CpGsites^c^** |  |  |  |  |  |  |
| Alcohol^a^  (N =34) | ADHFE1 | chr8:67344610-67344805 | 19 | 0.23 | 0.17 | 2.24×10^-1^ | 0.52 | 0.70 | 0.59 |
|  | EOMES | chr3:27764697-27764940 | 6 | 0.36 | 0.24 | 6.70×10^-3^ | 0.45 | 0.93 | 0.74 |
|  | SALL1 | chr16:51184268-51184468 | 17 | 0.35 | 0.20 | 2.75×10^-2^ | 0.45 | 0.96 | 0.68 |
|  | TFPI2 | chr7:93519367-93519503 | 19 | 0.30 | 0.14 | 1.30×10^-2^ | 0.55 | 0.93 | 0.71 |
|  | Combined | - | - | - | - | - | **0.74** | **0.78** | **0.77** |
| Non-alcohol^a^  (N = 58) | ADHFE1 | chr8:67344610-67344805 | 19 | 0.24 | 0.13 | 1.70×10^-3^ | 0.30 | 1.00 | 0.68 |
|  | EOMES | chr3:27764697-27764940 | 6 | 0.38 | 0.22 | 2.10×10^-7^ | 0.66 | 0.88 | 0.81 |
|  | SALL1 | chr16:51184268-51184468 | 17 | 0.37 | 0.17 | 3.00×10^-6^ | 0.57 | 0.94 | 0.78 |
|  | TFPI2 | chr7:93519367-93519503 | 19 | 0.27 | 0.13 | 2.90×10^-4^ | 0.55 | 0.86 | 0.71 |
|  | Combined | - | - | - | - | - | **0.66** | **0.96** | **0.84** |

^a^The Alcohol subgroup included the samples which had alcohol intake currently and formerly. ^b^Genomic region represents the genomic coverage of the reads with targeted bisulfite sequencing, and the genomic coordinates shown here were based on the hg19 version of the genome. ^c^No.CpGsites represents the number of the CpGsites in each region. ^d^McaM represents the mean methylation percentage of the cases in each region, which consists of several CpGsites, while McoM represents the mean methylation percentage of the controls in each region. ^e^P value is calculated through the Wilcoxon rank-sum test following with FDR (false discovery rate) adjustment for multiple correction. ^f^Sens is short for sensitivty, while Spec is short for specificity, and the AUC is short for area under curve. The sensitivity, specificity as well as the AUC were both with a logistic regression prediction model without adjustment for gender, age and smoking status and alcohol status. The mean methylation percentage of each in each genomic region was considered as a variable.

**Supplementary Figure 1 PCA analysis of the ESCC and EAC adjacent normal tissues.**

**Supplementary Figure 2 PCA analysis for the ESCC and adjacent normal tissues in the validation dataset**

**Supplementary Figure 3 The ROC (Receiver Operating characteristics) curve for the ESCC validation dataset**

**Supplementary Figure 4 The ROC (Receiver Operating characteristics) curve for the subgroup analyzes**

A-H represent the ROC curve for the prediction model in different subgroups, respectively. A-H each represent the overall ROC curve for the subgroup, which was calculated through a logistic regression model, incorporating the mean methylation percentage of the five genomic regions as the variables, and without the adjustment for gender, age and smoking status and alcohol status.
