# Supplementary figures and images for: Identification of Hyper-Methylated Tumor Suppressor Genes-Based Diagnostic Panel for Esophageal Squamous Cell Carcinoma (ESCC) in a Chinese Han Population
Source: Front Genet. 2018 Sep 5;9:356. doi: 10.3389/fgene.2018.00356 (PMC6133993; doi:10.3389/fgene.2018.00356)

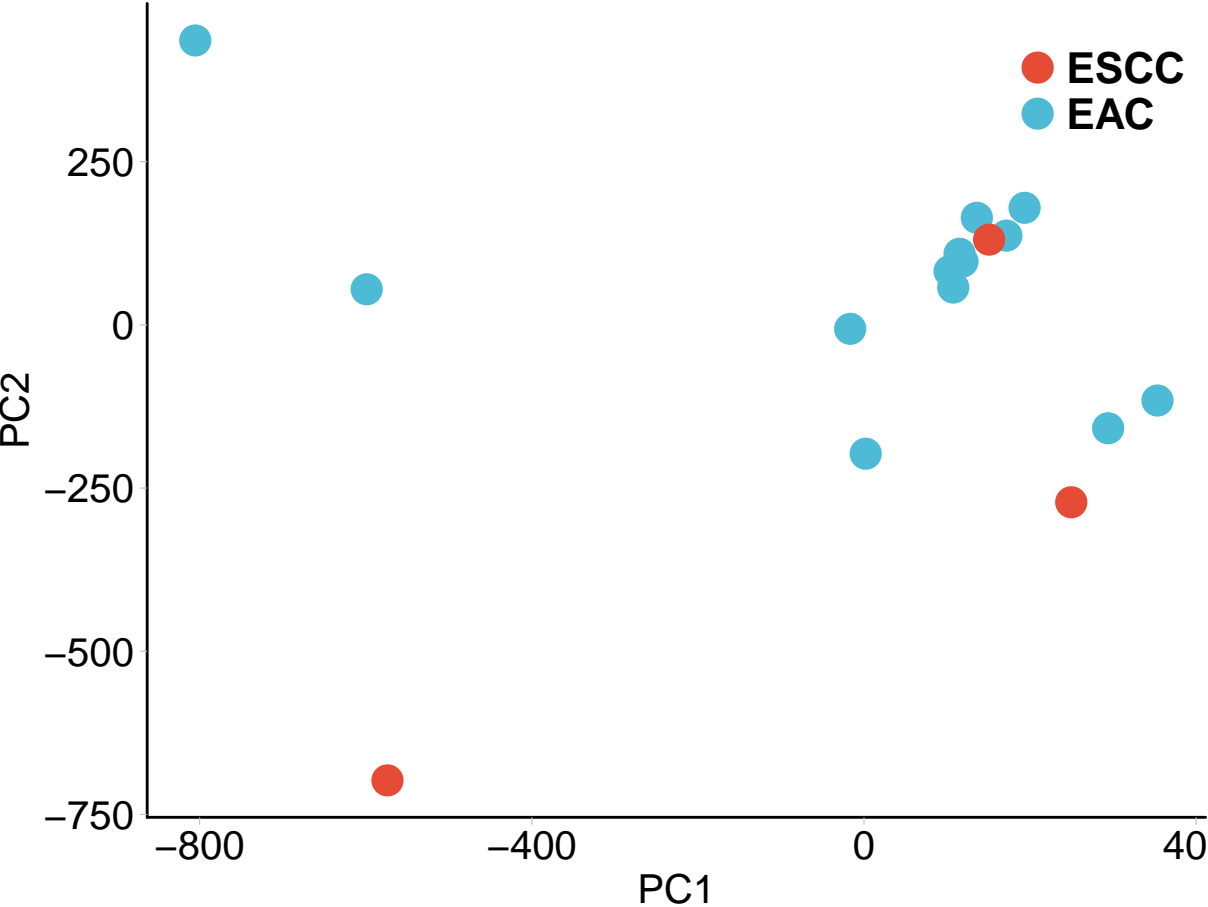

Supplement: Supplementary file 3 [file Image_1.PDF]

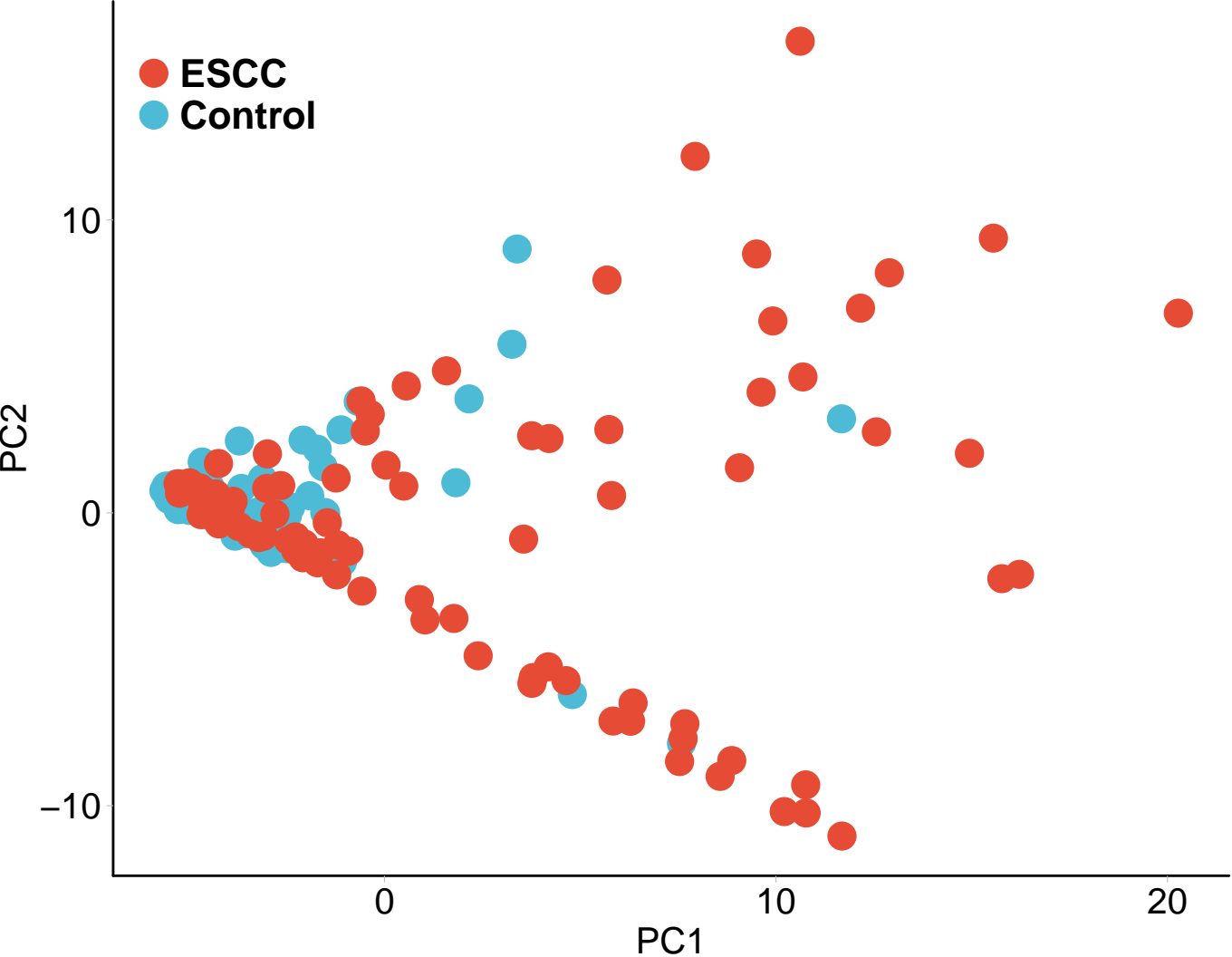

Supplement: Supplementary file 4 [file Image_2.PDF]

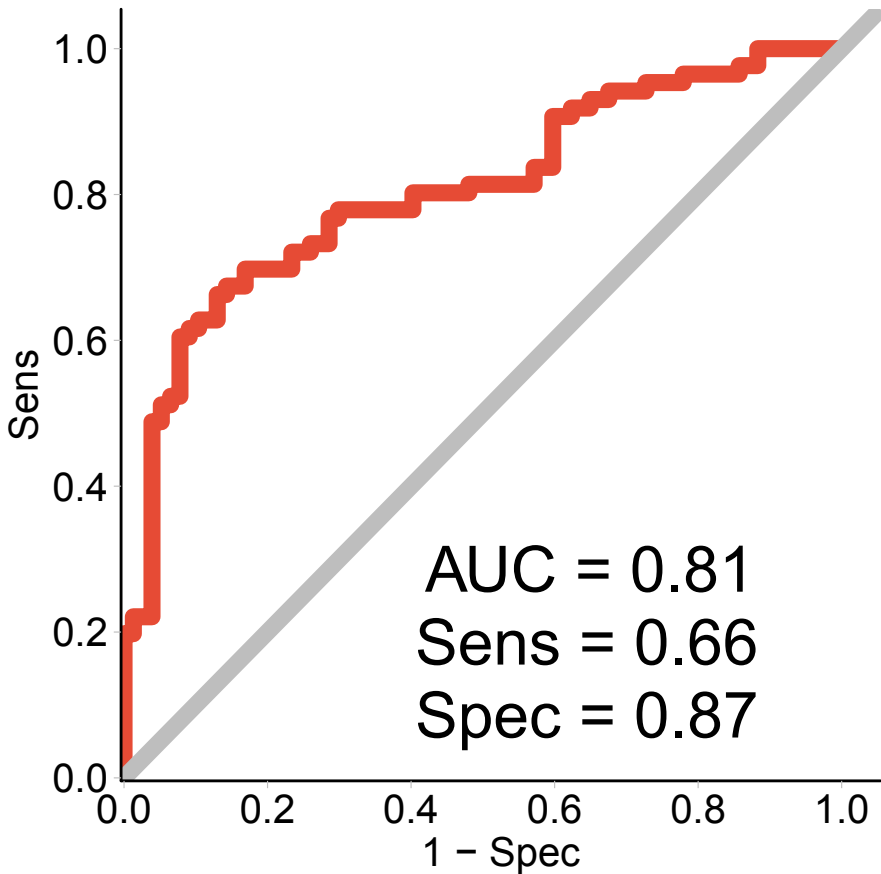

Supplement: Supplementary file 5 [file Image_3.PDF]

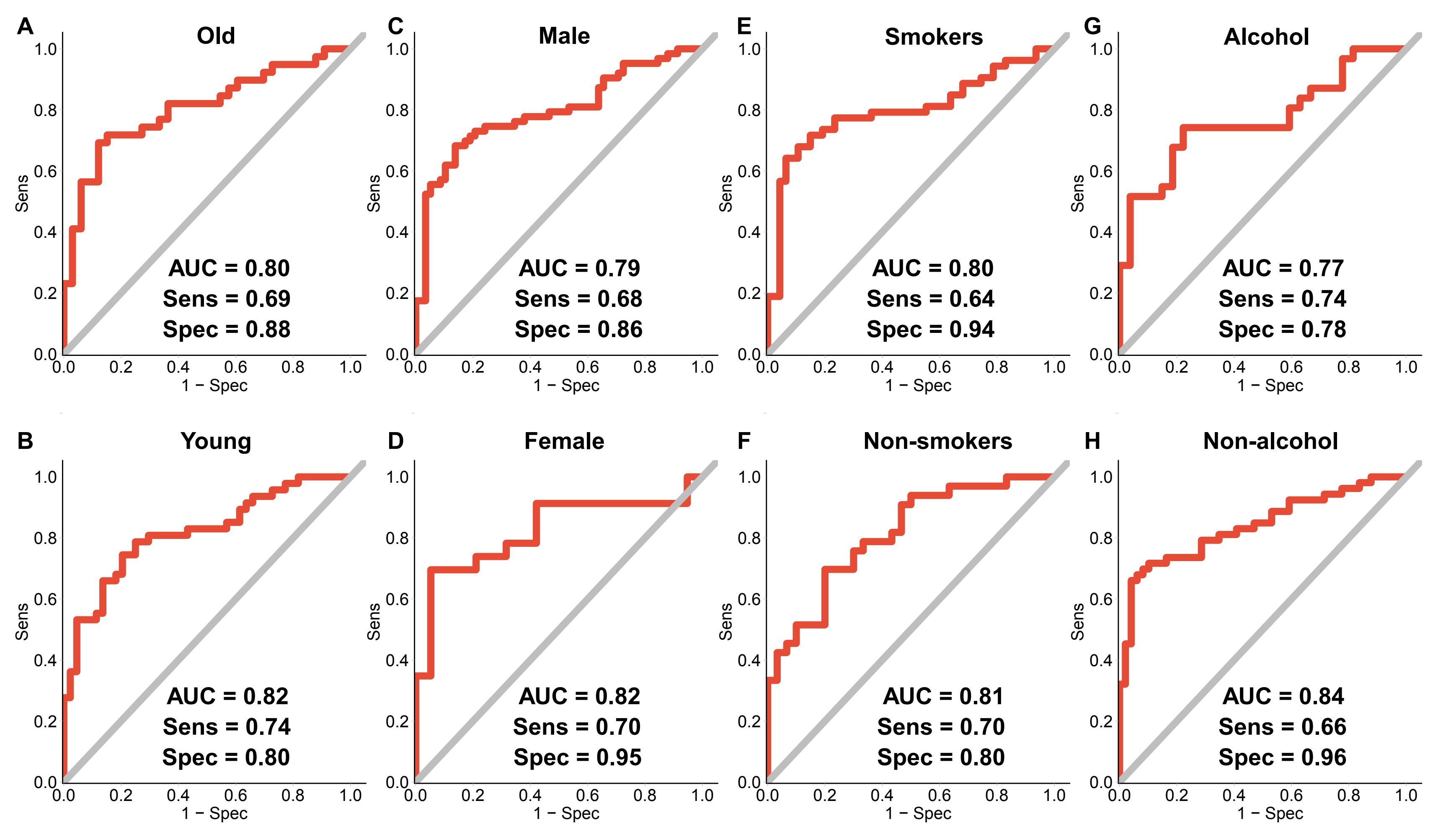

Supplement: Supplementary file 6 [file Image_4.PDF]
